# Deep learning-based fully automatic segmentation of the maxillary sinus on cone-beam computed tomographic images

**DOI:** 10.1038/s41598-022-18436-w

**Published:** 2022-08-17

**Authors:** Hanseung Choi, Kug Jin Jeon, Young Hyun Kim, Eun-Gyu Ha, Chena Lee, Sang-Sun Han

**Affiliations:** Department of Oral and Maxillofacial Radiology, Yonsei University College of Dentistry, 50-1 Yonsei-ro Seodaemun-gu, Seoul, 03722 Korea

**Keywords:** Dental diseases, Dentistry, Cone-beam computed tomography, Mathematics and computing

## Abstract

The detection of maxillary sinus wall is important in dental fields such as implant surgery, tooth extraction, and odontogenic disease diagnosis. The accurate segmentation of the maxillary sinus is required as a cornerstone for diagnosis and treatment planning. This study proposes a deep learning-based method for fully automatic segmentation of the maxillary sinus, including clear or hazy states, on cone-beam computed tomographic (CBCT) images. A model for segmentation of the maxillary sinuses was developed using U-Net, a convolutional neural network, and a total of 19,350 CBCT images were used from 90 maxillary sinuses (34 clear sinuses, 56 hazy sinuses). Post-processing to eliminate prediction errors of the U-Net segmentation results increased the accuracy. The average prediction results of U-Net were a dice similarity coefficient (DSC) of 0.9090 ± 0.1921 and a Hausdorff distance (HD) of 2.7013 ± 4.6154. After post-processing, the average results improved to a DSC of 0.9099 ± 0.1914 and an HD of 2.1470 ± 2.2790. The proposed deep learning model with post-processing showed good performance for clear and hazy maxillary sinus segmentation. This model has the potential to help dental clinicians with maxillary sinus segmentation, yielding equivalent accuracy in a variety of cases.

## Introduction

The maxillary sinus is the largest air-filled space around the nasal cavity and occupies most of the maxilla. It acts as a ventilation passage for incoming air through the nose^1^. The maxillary sinus begins to form as an invagination from the lateral wall of the middle meatus during fetal development and grows laterally and downward to the alveolar process at the age of 15–18 years. The inferior wall comprises the alveolar process, and the roots of premolars and molars are adjacent to or protrude into the maxillary sinus. Therefore, the maxillary sinus is highly relevant in the field of dentistry, and its evaluation and analysis are very important. Periapical lesions can cause sinusitis and an oroantral fistula may develop during tooth extraction procedures^1,2^. A sinus lift procedure may be required due to the lack of alveolar bone at the time of implant placement^3^. Conditions such as mucosal thickening of the maxillary sinus significantly affect the success rate and complications of implant surgery^4^. Patients with mucosal thickening greater than 50–75% of sinus volume should be referred to the otolaryngology department prior to surgery^5–7^. It is difficult to diagnose diseases affecting the maxilla early because the maxilla has multiple overlapping structures, so diseases are often discovered only after they are exacerbated^8^. Odontogenic cysts or tumors cause changes such as elevation of the sinus floor, and malignant tumors destroy the sinus floor. A close assessment of the maxillary sinus is helpful for making an early diagnosis.

Accurate segmentation (an anatomical annotation that delineates the outlines of important structures), is required as a cornerstone for automated diagnosis and treatment planning^9^ and constitutes the starting point for developing a model that segments only the area of mucosal thickening. When the maxillary sinus is segmented, its volume can be measured, thereby providing quantitative information on changes in maxillary sinus volume due to cysts and tumors. By providing the clinician with the volume ratio of the mucosal thickening area, accurate segmentation can offer guidance for sinus lift elevation during implant surgery.

Panoramic radiography is the most common imaging modality in dentistry^10^, but it has limitations in the accurate diagnoses of the maxillary sinus due to the presence of overlapping anatomical structures, such as the alveolar bone and zygomatic bone, and the geometric distortion of images. Cone-beam computed tomography (CBCT) has recently been used for the three-dimensional (3D) evaluation and diagnosis of oral and maxillofacial disease due to its advantages, which include a lower radiation dose, lower cost, and more convenient access compared with computed tomography (CT)^11^. CBCT provides 3D information through a multi-directional reconstruction process.

In clinical practice, manual segmentation by experts and semi-automatic segmentation are performed^12–14^. For an accurate analysis, multi-directional images must be considered, but doing so is laborious and time-consuming. Moreover, the results of the analysis may be highly dependent on experts’ knowledge and experience. For these reasons, deep learning^15^-based studies have been conducted with the goal of achieving equivalent accuracy in various cases, and several studies have made attempts to perform automatic segmentation of the maxillary sinus^16–18^. However, automatic segmentation of the maxillary sinus is challenging because it is connected to adjacent anatomical structures such as the frontal, ethmoid, and sphenoid sinuses, as well as nasal structures. Furthermore, automatic segmentation is even more difficult to perform on CBCT images because the quality of CBCT images is inferior to that of CT images due to image noise and poor soft tissue contrast^19,20^.

In recent studies, deep learning algorithms have shown good performance in automated detection, segmentation, and diagnosis based on medical images^21–23^. Various networks have been used for segmentation, and many studies have used U-Net algorithm^24–26^. Sinus segmentation has mostly utilized CT images^16,17^, whereas very few studies have analyzed CBCT images using deep learning^18^. We hypothesized that the maxillary sinus could be automatically and accurately segmented on CBCT images using a U-Net model with a post-processing algorithm to achieve similar accuracy to segmentation on CT images.

This study proposes a deep learning-based model for fully automated segmentation of maxillary sinus in various states (clear and various levels of haziness) using CBCT images and adds a post-processing step to the model for more accurate segmentation.

## Methods

### Datasets and image preparation

This study was approved by the Institutional Review Board (IRB) of Yonsei University Dental Hospital (NO. 2-2021-0059) and was conducted in accordance with relevant guidelines and ethical regulations. As a retrospective study, the requirement of patient informed consent was waived by the IRB of Yonsei University Dental Hospital. The data were anonymized to avoid identification of the patients.

**Figure 1:**
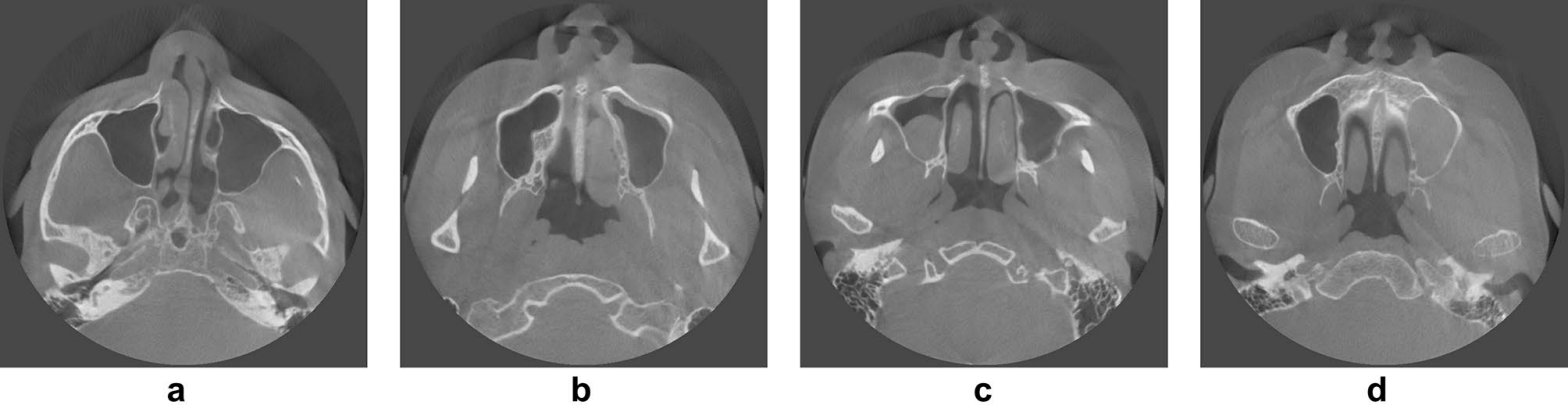
Examples of various maxillary sinus images. (**a**) Clear sinuses (both sides), (**b**) a slightly hazy sinus (left side), (**c**) a moderately hazy sinus (right side), (**d**) a severely hazy sinus (left side).

In total, 19,350 CBCT images were acquired from 90 maxillary sinuses of 45 patients (26 females and 19 males) who visited Yonsei University Dental Hospital from June 2020 to March 2021. There were 430 images per person, and the number of images including the sinus varied from 156 to 215. All CBCT data were acquired with a 16 × 10 cm field of view using a RAYSCAN Alpha Plus device (Ray Co. Ltd, Hwaseong-si, Korea). Cases with abnormalities or a history of surgery in the maxillary sinus were excluded, as were poor-quality images with artifacts. The 90 maxillary sinuses were clear or had various states of haziness, including mucosal thickening, mucous retention cysts, and fluid (Fig. 1). The datasets for training, validation, and testing were split into 6:2:2 ratios^27,28^. The characteristics of the subjects are summarized in Table 1.

**Table 1:** Number of maxillary sinuses in the datasets.

Sinus	Training	Validation	Testing
Clear	17	8	9
Hazy	37	10	9
Total	54	18	18

**Figure 2:**
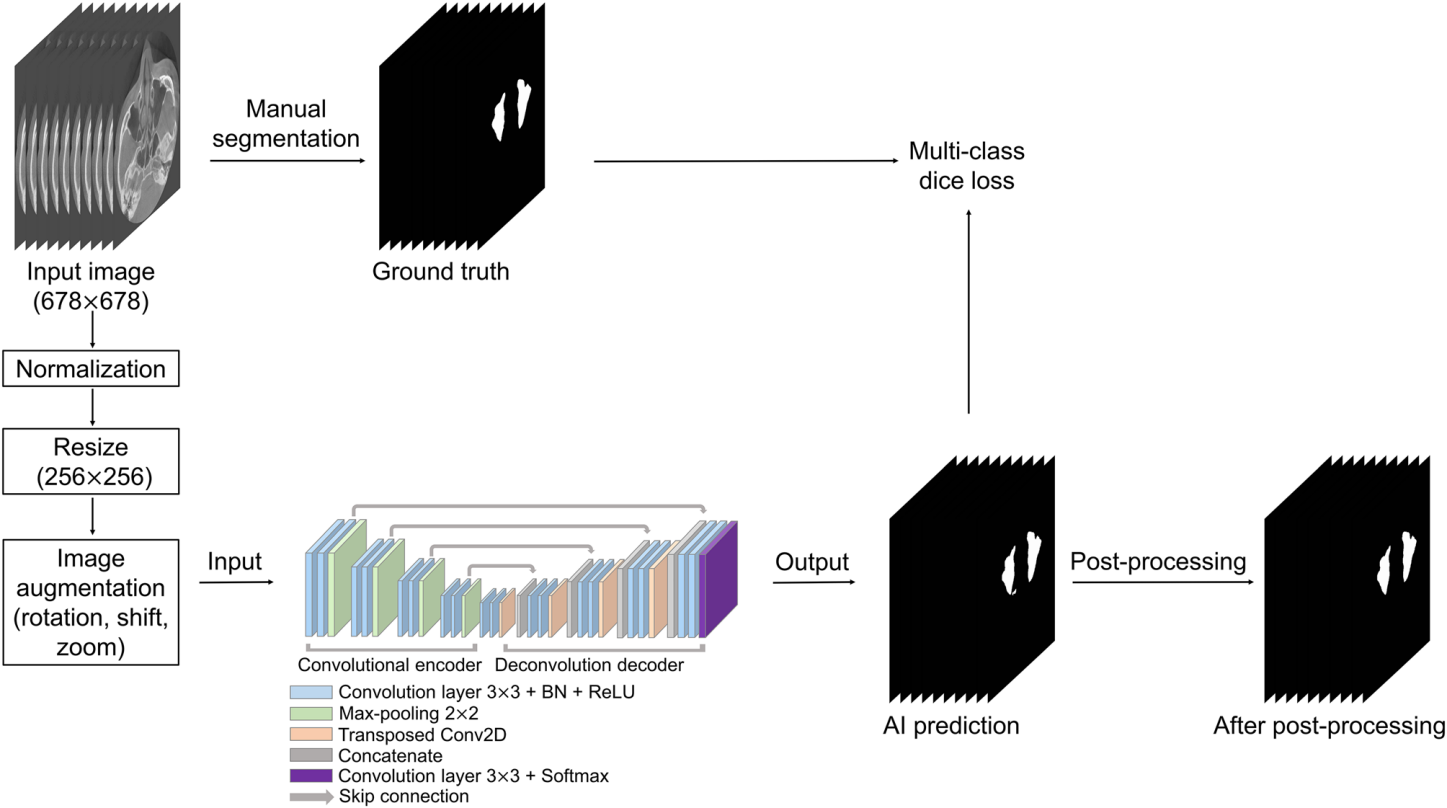
Overall process of our study.

CBCT data were used in the Digital Imaging and Communications in Medicine (DICOM) format with a matrix size of 678 (width) × 678 (height) pixels. To generate a label mask for the ground truth, an oral radiologist with over 20 years of experience conducted manual segmentation of the maxillary sinus in each axial image using 3D Slicer (free open-source software for biomedical image analysis)^29^. The label images were exported in the DICOM format and used as the ground truth for network training. Input images of 678 × 678 pixels were normalized by using two-dimensional min/max normalization and resized to 256 × 256 pixels. Data augmentation was performed with rotation (− 5° to 5°), translation shift (0–30%), and zoom (0–30%). Figure 2 shows the overall process.

### Network architecture

**Figure 3:**
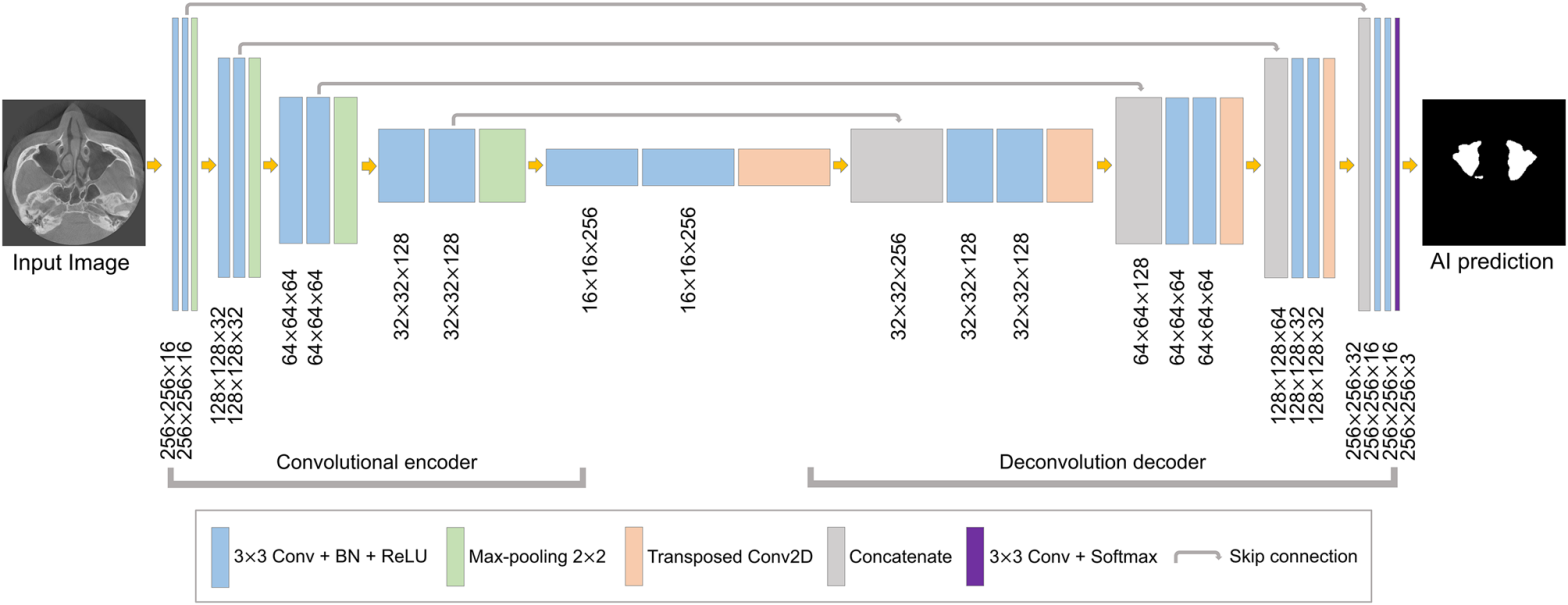
Structure of U-Net based convolutional neural network segmentation.

The segmentation model based on U-Net was designed for maxillary sinus segmentation. The U-Net model has demonstrated powerful performance in the segmentation of medical images^24–26^. This architecture is shown in Fig. 3. It consisted of a fully convolutional encoder and decoder, including 18 convolutional blocks, 4 max-pooling layers, 4 transposed convolution layers, and output layer. The convolutional block consisted of a 3 × 3 convolution with stride 1, batch normalization, and the ReLU activation function. At the end of the network, the softmax activation function in the output layer was used for multi-class segmentation of the background, right maxillary sinus, and left maxillary sinus. In the encoding block, the U-Net extracted the maxillary sinus features and passed them on to the next block. In the decoding block, the spatial information features were extracted, and then the size of feature maps was recovered to be the same as the input. The deep learning networks were implemented in Python 3 with the Keras library and trained on an NVIDIA Titan RTX (24 GB).

### Loss function

For training, the Adam optimizer^30^ was applied, and the learning rate was initially set to 10^−3^. While monitoring the loss of the validation set, if the validation loss did not decrease for 20 epochs, the training was stopped using an early stopper. The loss function of a deep learning network is calculated for validating the quantitative difference between the network output and ground truth. The network parameters are iteratively adjusted in the training phase to minimize loss. In order to train the proposed model, we employed the Dice coefficient^28^–based multi-class loss function (DL). The DL is defined as:

1 $$\mathrm{DL}\left(\mathrm{P},\mathrm{G}\right)=1-\frac{1}{K}\sum_{c=1}^{K}\frac{2\sum_{i}^{N}{p}_{i}^{c}{g}_{i}^{c}}{\sum_{i}^{N}{p}_{i}^{c}+\sum_{i}^{N}{g}_{i}^{c}}$$

where ${p}_{i}$ and ${g}_{i}$ represent the pixel values of prediction and ground truth, respectively, N is the number of pixels, and K is the number of classes (background, right maxillary sinus, and left maxillary sinus).

### Post-processing

**Figure 4:**
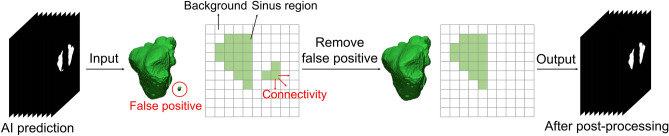
The post-processing workflow. False positives not connected to the maxillary sinus were eliminated.

Post-processing was performed to reduce the prediction errors of the developed U-Net model. If the maxillary sinus is adjacent to an air area such as the ethmoid sinus or connected to the airway, it tends to be incorrectly predicted. This intrinsic limitation of neural networks can lead to pixel-level prediction errors^31^, especially at maxillary sinus boundaries. To solve these problems, we conducted post-processing to eliminate false positives in the maxillary sinus segmentation results of the U-Net (Fig. 4). A 3D volume of the maxillary sinus can be obtained by stacking 2D slice images, and volume information can be acquired by using a connection component of a voxel. Background voxels are labeled with 0, and voxels of the maxillary sinus region are labeled with 1. Labels of 1 for small volumes not connected to the maxillary sinus were removed. Then, the 3D volumetric image was converted to 2D images. This method was implemented using MATLAB 2021a (MathWorks, Natick, MA, USA).

### Evaluation metrics

We used the dice similarity coefficient (DSC), precision, recall, and Hausdorff distance (HD)^32^ to evaluate the artificial intelligence (AI) prediction results and post-processing results. The DSC is the most frequently used evaluation method in medical image segmentation to compare a segmentation result R and the ground truth result G. The formula of the DSC is defined as:

2 $$\mathrm{DSC}=\frac{{2}\left|\mathrm{R}\cap \mathrm{G}\right|}{\left|{\text{R}}\right|+\left|{\text{G}}\right|}$$

To evaluate the segmentation quality, we used the precision and recall, which are defined as:

3 $$\mathrm{Precision}=\frac{\text{TP}}{{{\rm TP}} + {\text{FP}}}$$

4 $$\mathrm{Recall}=\frac{\text{TP}}{{{\rm TP}} \, + \, {\text{FN}}}$$

where TP, FP, and FN are true positive, false positive, and false negative, respectively. TP represents the number of pixels for which the maxillary sinus areas were accurately predicted. FP represents the number of pixels that were not maxillary sinus areas but were predicted to be maxillary sinuses. FN represents the number of unpredicted pixels in the maxillary sinus areas.

The HD measures the degree of difference between the two results by considering the spatial distance between the segmented objects as an index^33^. The closer the HD value is to 0, the more similar the prediction result is to the ground truth. The HD measures the maximum distance between two point sets, X and Y. The HD is defined as:

5 $${\text{HD}}(\mathrm{X},\mathrm{Y})=\max\left(\underset{x\in X}{{\max}}\,\underset{y\in Y}{{\min}}\,d\left(x,y\right),\underset{x\in Y}{{\max}}\,\underset{y\in X}{{\min}}\,d\left(x,y\right)\right)$$

where $x$ and $y$ denote two points between the ground truth and prediction result of the maxillary sinuses, and $d\left(x,y\right)$ is the distance between the two points.

## Results

**Figure 5:**
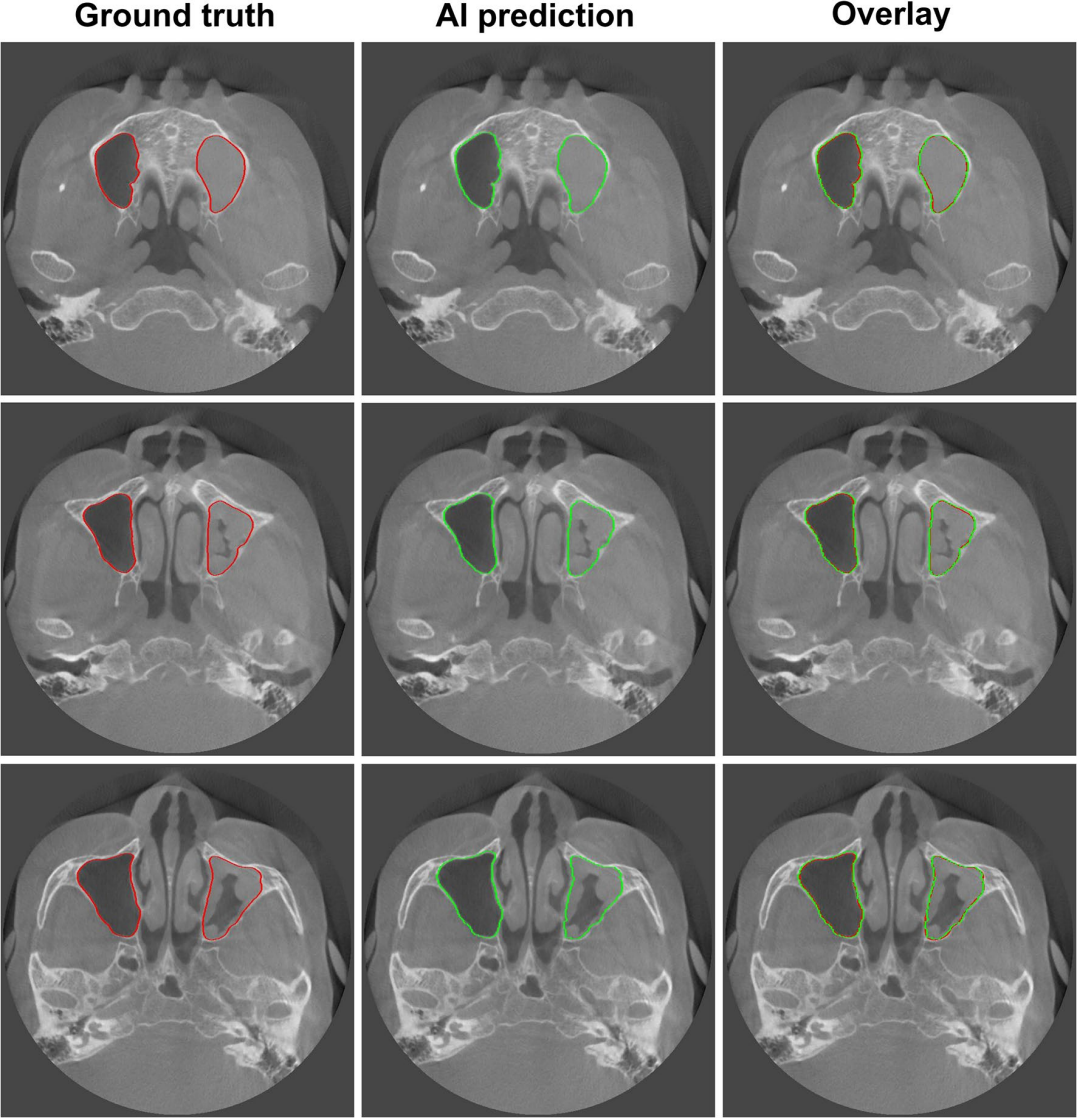
Best results of maxillary sinus segmentation. The right side presents a clear maxillary sinus, and the left side shows a hazy maxillary sinus. Red: the edges of the ground truth image; green: the edges of the artificial intelligence (AI) prediction image.

**Figure 6:**
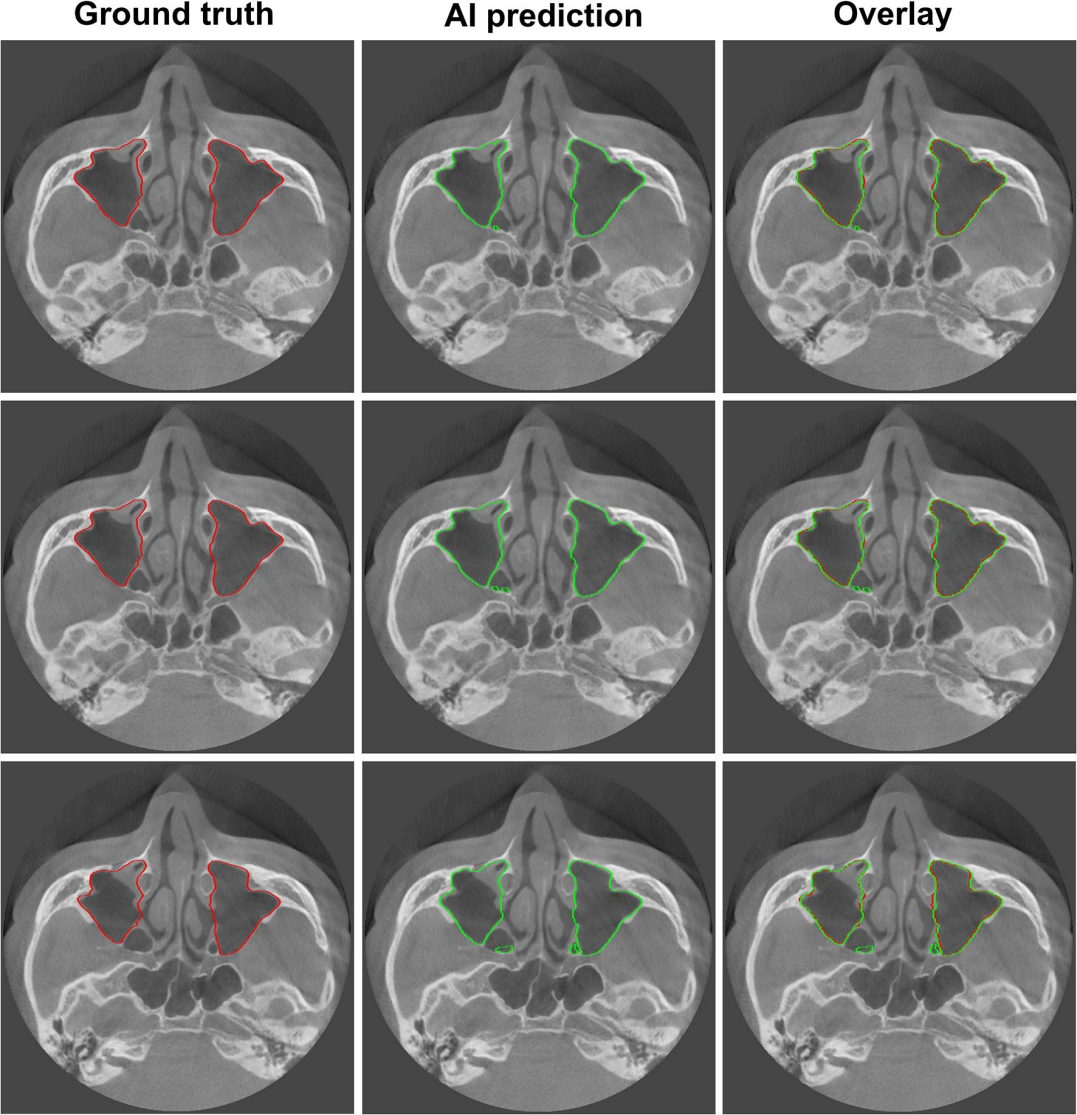
Worst results of maxillary sinus segmentation. The mispredicted part in the posterior part of the maxillary sinus is the ethmoid sinus. Red: the edges of the ground truth image; green: the edges of the artificial intelligence (AI) prediction image.

**Figure 7:**
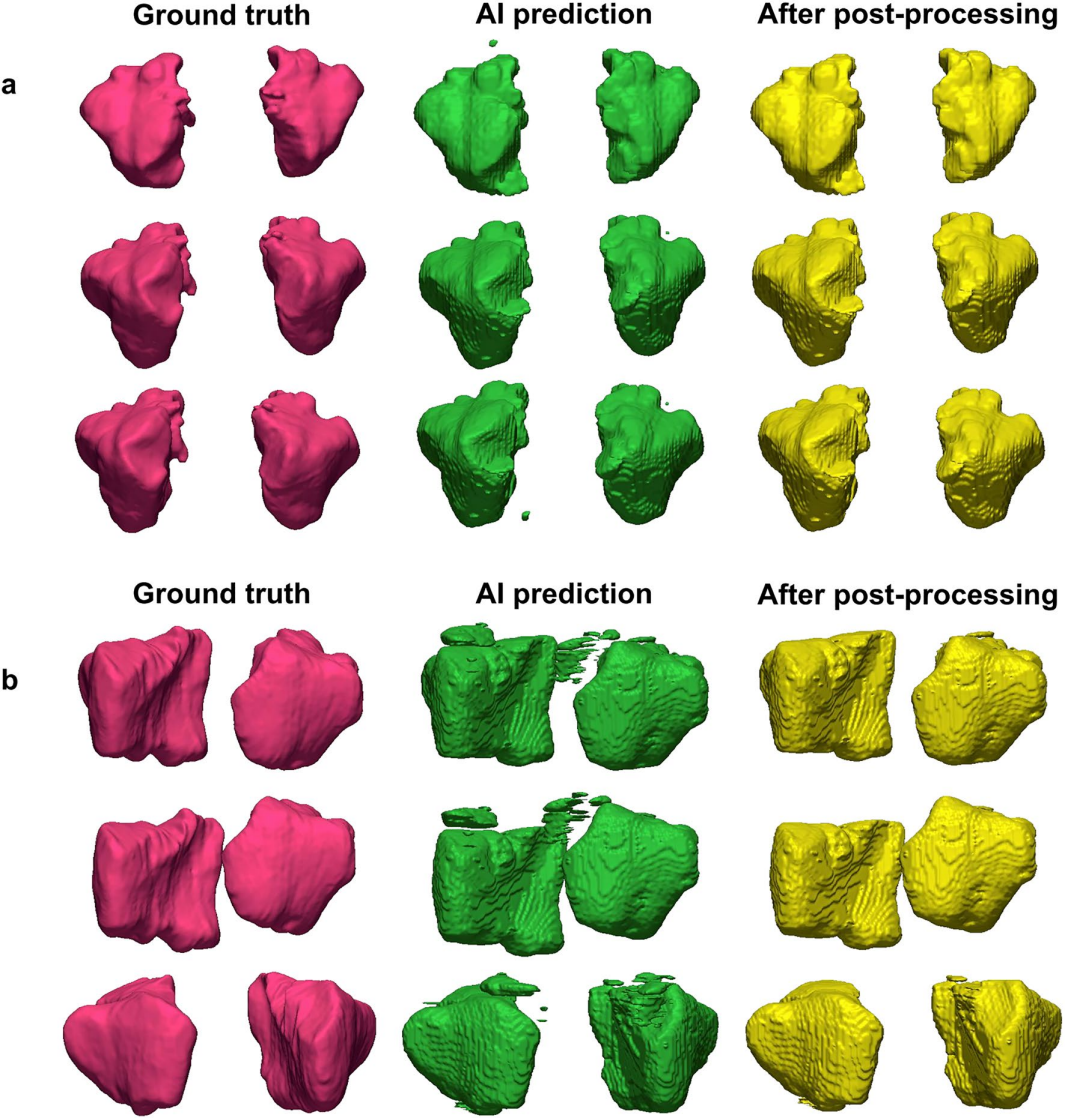
Three-dimensional reconstruction results. (**a**) Clear maxillary sinus, (**b**) hazy maxillary sinus. Red: the ground truth (manual labels); green: the results of artificial intelligence (AI) prediction; yellow: the results after post-processing of AI prediction. The average segmentation time per maxillary sinus was 48.7 min for the manual method and 46.2 s for the AI method, and the post-processing process took an average of 16 s.

Figures 5 and 6 show the best and worst results of AI prediction for maxillary sinus segmentation. The 3D reconstruction results of AI prediction and after post-processing are shown in Fig. 7. False positives were removed in post-processing. The average prediction results of AI prediction and after post-processing are shown in Table 2. The DSC of the AI model was 0.9090 ± 0.1921 and the HD was 2.7013 ± 4.6154. After post-processing, the DSC was 0.9099 ± 0.1914 and the HD was 2.1470 ± 2.2790. Table 3 shows the final AI prediction results after post-processing according to sinus conditions. The average segmentation time per maxillary sinus was 46.2 s for the AI method, whereas it took 48.7 min for the manual method, and the post-processing process took an average of 16 s.

**Table 2:** Prediction results of AI prediction and after post-processing.

	AI prediction (mean ± SD)	After post-processing (mean ± SD)
DSC	0.9090 ± 0.1921	0.9099 ± 0.1914
Precision	0.8977 ± 0.1874	0.8999 ± 0.1860
Recall	0.9282 ± 0.2005	0.9282 ± 0.2005
HD (mm)	2.7013 ± 4.6154	2.1470 ± 2.2790

*AI* artificial intelligence, *DSC* dice similarity coefficient, *HD* Hausdorff distance, *SD* standard deviation.

**Table 3:** Final AI prediction results after post-processing according to sinus condition.

	Clear sinus (mean ± SD)	Hazy sinus (mean ± SD)
DSC	0.9072 ± 0.1842	0.9123 ± 0.1977
Precision	0.8852 ± 0.1784	0.9131 ± 0.1917
Recall	0.9404 ± 0.1935	0.9173 ± 0.2060
HD (mm)	2.4057 ± 2.4496	1.9158 ± 2.0887

*AI* artificial intelligence, *DSC* dice similarity coefficient, *HD* Hausdorff distance, *SD* standard deviation.

## Discussion

The present study proposed a CNN model for the fully automatic segmentation of various maxillary sinuses on CBCT images. The inferior wall of the maxillary sinus is part of the alveolar process, making it highly relevant for dentistry (e.g., tooth extraction, implants, and apical lesions). When a disease such as a cyst or a tumor occurs in the maxilla, the change in the maxillary sinus is an important aspect of the diagnosis, and evaluation of the maxillary sinus is also important in sinus lift procedures as part of implant surgery. Therefore, automatic maxillary sinus segmentation will play a starting point for clinical diagnoses and quantitatively measuring volume. By providing the volume ratio of the mucosal thickening area, automatic segmentation will be able to offer guidance for referral to an otolaryngologist before sinus lift elevation for implant surgery.

The maxillary sinus is connected to adjacent anatomical structures, which are very difficult to separate accurately. Inflammation of the sinus appears in various forms, such as thickening of the mucous membrane. If there is mucosal thickening, the border is not clearly defined, which may make automatic segmentation more difficult. A few studies have explored the application of automatic maxillary sinus segmentation using CT images^16,17^. Xu et al.^16^ used an improved V-Net to segment the maxillary sinus and reported that the DSC value was 0.94 in clear maxillary sinuses. However, it has been reported that the accuracy was still unsatisfactory in cases of mucosal inflammation. In another study, Iwamoto et al.^17^ developed a maxillary sinus segmentation model using a fully convolutional network with a probability atlas, and the DSC value was reported to be 0.83 in hazy maxillary sinuses.

CBCT is widely used in dental clinics because it has several advantages over traditional CT and panoramic radiography. It provides 3D information, unlike panoramic radiography, and it has a significantly lower radiation dose and substantially lower costs than CT. CBCT is often used to diagnose the condition of the maxillary sinus and for planning extraction and implant placement in the maxilla. To the best of our knowledge, only one study has performed automatic maxillary sinus segmentation using CBCT images^18^. Jung et al.^18^ segmented the air area and the lesion area in the maxillary sinus and obtained DSCs of 0.93 and 0.76, respectively. However, the results of the entire maxillary sinus segmentation were not shown. It seems more reasonable to accurately segment the maxillary sinus first and then segment the air and the lesion area. Hazy sinuses are particularly important because they are associated with a higher likelihood of complications after tooth extraction or implantation, but the performance of segmentation is worse for hazy sinuses than for clear sinuses. Therefore, we added more data on hazy sinuses, and the model performance was good, with a DSC value of 0.9 or higher for both clear and hazy sinuses.

In this study, we proposed a deep learning-based method using a U-Net model with post-processing. The non-maxillary sinus area was incorrectly predicted as the sinus area in some cases, and post-processing was used to reduce these false-positive errors. After post-processing, the average DSC value increased from 0.9090 ± 0.1921 to 0.9099 ± 0.1914 and the average HD value decreased from 2.7013 ± 4.6154 to 2.1470 ± 2.2790. The change in the predicted area due to the removal of false positives was small, but the HD value considerably improved. Our U-Net with post-processing method showed similar or higher performance in comparison with previous CT studies^16,17^. In our model, there is no need for an expert to manually select the maxillary sinus from all images for segmentation. We automatically and accurately segmented the maxillary sinus from CBCT images within 1 min, which was much more efficient than manual segmentation, which took more than 30 min. The developed model makes it possible to analyze the CBCT images of many patients in a short time.

Although our study showed good performance for automatic segmentation of the maxillary sinus, some limitations should be addressed in future research. First, if false positive pixels are connected to the maxillary sinus region, they are not removed by post-processing. To overcome this limitation, a process for detecting the edge of the maxillary sinus is required. Using a variety of training data may also help solve this problem. Second, this study was conducted using a small sample size of 45 patients (90 maxillary sinuses), and data were obtained using a single CBCT device. Using more images from various CBCT devices will improve the performance of the algorithm. In further study, developing a segmentation model of areas of only haziness is needed. A model that could segment only the hazy area of the maxillary sinus and measure the ratio of the air area and hazy area of the maxillary sinus would be helpful in planning surgery or treatment.

## Conclusion

The developed deep learning algorithm with post-processing showed good performance for both clear and hazy maxillary sinus segmentation with minimal time and effort. The proposed model has the potential to assist dental clinicians in segmenting the maxillary sinuses in various cases with equivalent accuracy in CBCT images.

## Data Availability

The data generated and analyzed during the current study are not publicly available due to privacy laws and policies in Korea but are available from the corresponding author on reasonable request.
